# Embryonic, Larval, and Early Juvenile Development of the Tropical Sea Urchin, *Salmacis sphaeroides* (Echinodermata: Echinoidea)

**DOI:** 10.1100/2012/938482

**Published:** 2012-09-27

**Authors:** M. Aminur Rahman, Fatimah Md. Yusoff, A. Arshad, Mariana Nor Shamsudin, S. M. N. Amin

**Affiliations:** ^1^Laboratory of Marine Biotechnology, Institute of Bioscience, Universiti Putra Malaysia, 43400 UPM Serdang, Selangor, Malaysia; ^2^Department of Aquaculture, Faculty of Agriculture, Universiti Putra Malaysia, 43400 UPM Serdang, Selangor, Malaysia

## Abstract

*Salmacis sphaeroides* (Linnaeus, 1758) is one of the regular echinoids, occuring in the warm Indo-West Pacific, including Johor Straits, between Malaysia and Singapore. In order to investigate the developmental basis of morphological changes in embryos and larvae, we documented the ontogeny of *S. sphaeroides* in laboratory condition. Gametes were obtained from adult individuals by 0.5 M KCl injection into the coelomic cavity. Fertilization rate at limited sperm concentration (10^−5^ dilution) was 96.6 ± 1.4% and the resulting embryos were reared at 24°C. First cleavage (2-cell), 4-cell, 8-cell, 16-cell, 32-cell, and multicell (Morulla) stages were achieved 01.12, 02.03, 02.28, 02.51, 03.12, and 03.32 h postfertilization. Ciliated blastulae with a mean length of 174.72 ± 4.43 **μ**m hatched 08.45 h after sperm entry. The gastrulae formed 16.15 h postfertilization and the archenteron elongated constantly while ectodermal red-pigmented cells migrated synchronously to the apical plate. Pluteus larva started to feed unicellular algae in 2 d, grew continuously, and finally attained metamorphic competence in 35 d after fertilization. Metamorphosis took approximately 1 h 30 min from attachment to the complete resorption of larval tissues and the development of complete juvenile structure with adult spines, extended tubefeet and well-developed pedicellaria, the whole event of which usually took place within 1 d postsettlement. This study represents the first successful investigation on embryonic, larval, and early juvenile development of *S. sphaeroides*. The findings would greatly be helpful towards the understanding of ontogeny and life-history strategies, which will facilitate us to develop the breeding, seed production, and culture techniques of sea urchins in captive condition.

## 1. Introduction

*Salmacis sphaeroides* (Linnaeus, 1758) (Echinodermata: Echinoidea: Temnopleuridae), or ball-like white sea urchin, is one of the regular echinoids, occuring most abundantly in the warm Indo-West Pacific where it can be found from China to Solomon Islands and Australia [1, 2], and Singapore [3]. It can also be found in the warm temperate regions including Johor Straits, between Malaysia and Singapore [3]. This sea urchin can occurr at depth ranging between 0 and 90 m, however it is generally found in shallow waters, especially in amongst seagrass meadows and in muddy sublittoral zone or washed ashore [3]. It has almost cloudy white test (5.0 to 8.0 cm diameter) with numerous short spade-like spines (1.0 to 1.5 cm long). Some may have white spines with maroon bands, others with all maroon spines, and yet others with green and maroon bands. This species is also recognized to inhabit in shallow seagrass bed and coral reef areas [1]. *Salmacis sphaeroides* gets their food from algae, bryzoans, seaweeds, and detritus [2]. This behavior shows that the animal is an omnivorous scavenger and detritus feeder, ingesting loose substrates and scraping films off hard surfaces and that is why it can also be found on algal substrates [4].

Sea urchins are classic objects of research in different fields of biology and ecology. At the same time, they are used as raw material to produce foodstuff, in particular, the product of processing gonads known as “Sea urchin Roe or Uni” [5–7] and are considered a prized delicacy in Asia, Mediterranean countries, and Western Hemisphere countries such as Barbados and Chile [8]. People of the Asian Pacific Region have also used sea urchin gonads for many years as a remedy for improving general body condition, treatment for a number of diseases, and strengthening of sexual potency of men [9]. Gonads of sea urchins have long been a luxury food in Japan [10]. Although, *S. sphaeroides* has not yet been used as edible species in Malaysia, it has been found to serve as a delicacy food item in local seafood restaurants in Hong Kong [11]. 

Sea urchin gonads are also rich in valuable bioactive compounds, such as polyunsaturated fatty acids (PUFAs) and **β**-carotene [12]. PUFAs, especially eicosapentaenoic acid (EPA, C20:5) (n-3)) and docosahexaenoic acid (DHA C22:6 (n-3)), have significant preventive effects on arrhythmia, cardiovascular diseases, and cancer [13]. **β**-Carotene and some xanthophylls have strong provitamin A activity and can be used to prevent tumour development and light sensitivity [14]. On the other hand, the high levels of AA and EPA recently detected in *S. sphaeroides *supported the development of aquaculture of this urchin [11], since PUFAs are important for human nutrition [15]. Sea urchin fisheries have expanded so greatly in recent years that the populations of sea urchins around the world have been overfished [16, 17]. Not surprisingly, the decrease in supply and the continued strong demand have led to a great increase in interest in aquaculture of sea urchins, particularly in those areas where their populations have been depleted [8, 18, 19]. 

Considering the enormous importance of *S. sphaeroides*, early life history information is an essential requirement for optimization of large scale seed production, culture, and management. A few studies on its distribution and feeding ecology have recently been carried out [20–23], but no systematic studies have yet been conducted to optimize larval growth and survival. Therefore, an attempt was made to study the detailed embryonic and larval development of *S. sphaeroides* in a controlled lab-rearing condition. 

## 2. Materials and Methods

### 2.1. Sample Collection and Maintenance

Mature adults of the sea urchin, *S. sphaeroides*, weighing from 90 to 200 g, were collected from Merambong shoal off Tanjung Kupang (01°34′N; 103°60′E), Johor at low tide during their natural breeding season from April to July, 2011. Immediately after collection, the live sea urchins were transported to the Laboratory of Marine Biotechnology, Institute of Bioscience, Universiti Putra Malaysia, where they were maintained in aerated closed aquaria before use for the experiments. 

### 2.2. Spawning and Fertilization

Most of the urchins were used for this experiment within a week after collection. The Aristotle's lantern was removed from the healthy specimens by using scissors and forceps and rinsed thoroughly with filtered sea water (FSW). Gametes were obtained from each sea urchin following the injection of 0.5 M KCl solution into the coelomic cavity. Eggs were collected by inverting female urchins over a glass beaker filled with filtered sea water (FSW). “Dry” sperm were pipetted off the genital pores and kept in concentrated form in a refrigerator at 4-5°C for not more than 3-4 h. Diameter of eggs and length of sperm head were measured (eggs at 20 × 10 in a well-slide, sperms at 40 × 10 on a plain slide) by a compound microscope, using the methods of Rahman et al. [24, 25]. Fertilization was done by mixing two drops of a diluted sperm into a petri dish containing 15 mL egg suspensions. The sperm concentration was maintained at 10^−5^ dilution of “dry” sperm [26, 27]. Sperms were then allowed to remain with the eggs for 5–10 minutes and then excess sperms were removed by 3-4 consecutive washes with SFSW. Six replicate fertilization experiments were performed using fresh gametes from new individuals in each time. The first 100 eggs encountered were classified as “fertilized” if they had reached the 2–4 cell stage [27, 28].

### 2.3. Embryonic and Larval Development

The fertilized eggs were transferred to 500-mL glass beakers and incubated in FSW at ambient room temperature (24°C) until they attained free swimming blastula stage. They were then transferred to glass bottles containing 500 mL FSW, which was stirred constantly by 10 rpm rotating motors. Larval densities up to the four-armed pluteus stage were maintained at 2-3 individuals/mL, following the methods described by Rahman et al. [26, 27]. When the larvae attained feeding stage (four-armed pluteus), they were cultured in the same system (500 or 1000 mL glass bottles with a larval density of 1 individual/mL). About 90% of the culture water was removed by filtration/siphoning every 4-5 days and replaced with fresh FSW. Larvae were supplemented with a laboratory cultured phytoplankton, *Chaetoceros calcitrans *at concentrations of 5000, 10,000, and 15,000 cells per mL of medium at four-, six- and eight-armed stage, respectively, by adjusting the food level every 3 days until attaining metamorphic competence [26]. All the developmental stages of embryos and larvae were observed at time intervals after insemination until they reached metamorphic competence. At each stage, specimens were fixed in 10% formalin for more detailed studies. Observations on both living and fixed specimens, provided information on the times required for embryos to attain specific developmental stages. In each experiment, the times after insemination for 50% of the embryos to develop to 2-cell, 4-cell, 8-cell, blastula, gastrula, prism, 2-, 4-, 6-, 8-armed pluteus and competent stages were estimated, following Rahman et al. [24] and Fujisawa [29].

### 2.4. Induction of Metamorphosis

When the matured larvae deemed competent, were then used for settlement induction. Competence was indicated by the presence of large juvenile rudiments and a high rate of metamorphosis. Induction of metamorphosis was performed on coralline red algal extracts + *Chaetoceros *diatom (50 : 50) in the petri dishes (9.0 × 3.0 cm) containing FSW. Larval density at this stage was maintained at 1 individual/2 mL FSW following the method of Rahman and Uehara [30]. In each experiment, replicate petri dishes were used and metamorphosis rate was estimated within 24–30 h in the same environmental conditions and protocols as larval cultures.

### 2.5. Morphometric Measurements

All morphometric measurements of embryo, larvae, and newly metamorphosed juveniles were made on freshly prepared specimens, following Rahman et al. [25] and McEdward [31] with slight modifications. Larvae were killed in 10% formalin in FSW and were concentrated by settling to the bottom of a vial. A few drops of formalin-seawater containing 10–12 larvae were put under an elevated coverslip on a microscope slide. After that, it was observed and finally measured and photographed under the compound microscope (Zeiss Axioskop 2) fitted with a software (Spot Advanced Verson 3.4). Each sample was observed four times to identify the developmental stages [32].

## 3. Results

### 3.1. Embryonic Development

**Figure 1:**
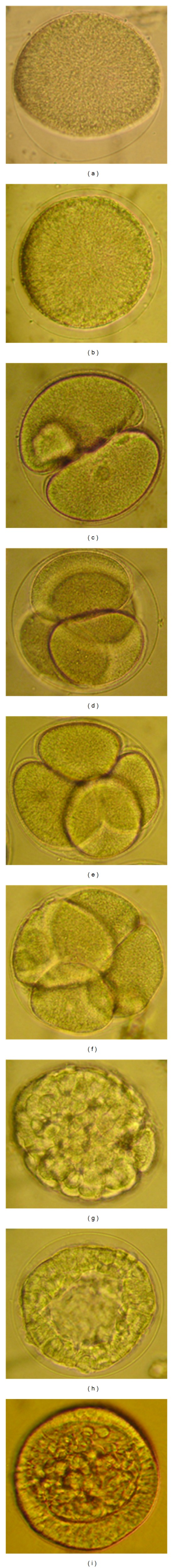
Embryonic developmental stages of *S. sphaeroides* under compound microscopy. (a) Fertilized egg showing fertilization membrane, (b) Fertilized egg with complete fertilization membrane (c) 2-cell stage, (d) 4-cell stage, (e) 8-cell stage, (f) 16-cell stage, (g) 32-cell stage, (h) Morulla stage enclosed with fertilization membrane, (i) Blastula.

The morphological events occurring during the embryonic development of *S. sphaeroides *are depicted in Table 1, while the developmental stages are shown in Figure 1.

**Spawning and Fertilization**

Sexually matured adult males and females of *S. sphaeroides* have 5 gonopores at the aboral side of the test. During spawning by intercelomic injection with KCl solution, the gonopores released gametes continuously for several minutes (maximum of 30 min for female and 15 min for male). The diameter of the unfertilized eggs of *S. sphaeroides *was 122.63–138.26 *μ*m (mean ± SD = 129.88 ± 4.40 *μ*m, *n* = 30). The matured eggs are transparent, spherical in shape, nonadhesive, yellowish in color and devoid of oil globules. The head length of matured sperm was 4.47–6.84 *μ*m (mean ± SD = 5.67 ± 0.61 *μ*m, *n* = 30) and was whitish in color. At limited sperm concentration (10^−5^ dilution of “dry” sperm), fertilization rate was 94.0–98.0% (mean ± SD = 96.6 ± 1.4%, *n* = 6). The egg vitelline membrane was elevated after 30–40 sec of sperm entry and the fertilization membrane began to form (Figure 1(a)). However, the complete formation of fertilization envelope took place within 5 min after insemination (Table 1; Figure 1(b)). Upon sperm penetration, the male pronucleus was pushed forward by microtubules towards the centre of the egg. When touched by microtubules, the female pronucleus was rapidly pulled towards the male pro-nucleus. This sperm-egg fusion took place approximately 10–12 min after sperm entry. During the fertilization proceedings, the cytoplasmic movements increased and the cell surface acquired an irregular aspect. Immediately before the starting of the first cleavage, the membrane ceased the vibration, the cell surface became regular, and the hyaline layer thickened.

**Cleavages**

First cell division started 01.12 h after fertilization (Table 2) and was holoblastic (Figure 1(c)). Second cleavage initiated 02.03 h after fertilization (Table 1) and was meridional, dividing the embryo into 4 equal blastomeres (Figure 1(d)). The third cleavage was equatorial, separating animal and vegetal blastomeres at 02.28 h (Table 1; Figure 1(e)). During the 4th division, micromeres originated equally from vegetal blastomeres while 8 mesomeres were formed by a meridional cleavage of animal blastomeres (Figure 1(f)) at 02.28 h after fertilization (Table 1). Equatorial division of mesomeres, meridional division of macromeres, and unequal micromere division formed embryos with 32 cells 03.12 h after fertilization (Table 1; Figure 1(g)). Sixty four-cell embryos were formed when blastomeres went through an equatorial division while the micromeres experienced a meridional division. The seventh cleavage occurred without micromere division and the embryos formed Merulla with 108 cells after 03.32 h following fertilization (Table 1; Figure 1(h)).

**Blastula**

Cells acquired a polygonal shape during the consolidation of the epithelium. The vegetal plate thickened and cilia were formed on the perimeter 08.45 h after fertilization, immediately before hatching (Table 1; Figure 1(i)).

### 3.2. Larval and Early Juvenile Development

**Figure 2:**
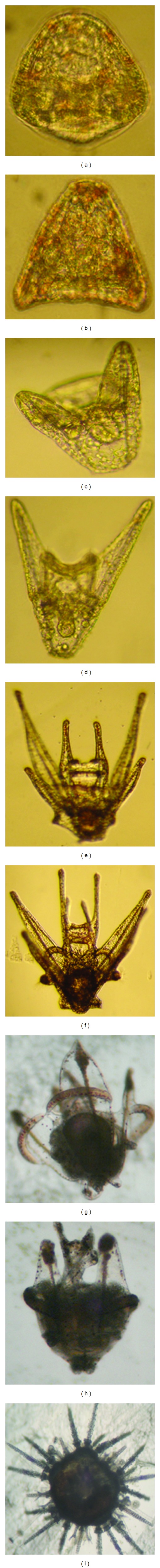
Larval developmental stages of *S. sphaeroides* under compound microscopy. (a) Gastrula, (b) Prism, (c) 2-arm pluteus, (d) 4-arm pluteus, (e) 6-arm pluteus, (f) 8-arm pluteus, (g) Pre-competent larva with ciliated ring and growing rudiment, (h) Competent larva with complete rudiment growth, (i) Juvenile (1 d after metamorphosis).

The morphological changes occurring during the larval an early juvenile development of *S. sphaeroides *are depicted in Table 1, while the developmental stages are shown in Figure 2.

**Grastrula**

The ciliated gastrula was formed 16.15 h after fertilization (Table 2). At the beginning of this stage, larva experienced with primary mesenchyme cells (PMC) which were detached from the vegetal pole, became spherical, and aggregated in a unipolar manner on the vegetal pole. PMC then migrated through the blastocoel forming a ring connected by thin pseudopodia on the posterior end. In this stage, red-pigmented cells were first observed on the vegetal pole and then migrated through the epithelium, simultaneously with PMC, towards the apical plate. PMC formed two aggregates and initiated the secretion of a calcareous triradiate spicule. Secondary mesenchyme cells (SMC) originated on the vegetal pole, extending cytoplasm projections towards the blastocoel during archenteron invagination. SMC on the archenteron then reached the anterior pole while red-pigmented epithelial cells reached the anterior pole, when the blastocoel was occupied by SMC (Figure 2(a)).

**Prism**

Prism stage initiated 22.25 h after fertilization. (Table 2). Epithelial red-pigmented cells were not present on the ventral (oral) region of the embryo at prism stage. During the course of the complete development of prism larva, the surface of the embryo was covered by cilia with an apical tuft on the anterior pole and a ciliated ring around the anus (Figure 2(b)).

**Pluteus**

The 2-arm pluteus stage was formed 34.00 h after fertilization (Table 2; Figure 2(c)). At this stage the mouth opened, but the larvae were unable to feed; microalgae captured by the larval arms were carried towards the mouth, but were deflected away possibly by an opposing current. The gut already had three portions identified as esophagus, stomach, and intestine but was not functional. Muscles of the esophagus began to contract; the stomach grew in diameter while its epithelium became thinner.

In 48.00 h after fertilization, 4-arm pluteus larva was formed with two well-developed postoral arms (Table 2; Figure 2(d)). At this stage, pluteus larva experienced with complete digestive tract and capable of feeding unicellular algae. The well-defined opening in the lower half of the larva represented the anus. The tips of arms and the arched oral lobe behind them represented the leading front of the swimming larva under the oral lobe which directed algae into the mouth. During the onset of the 6-arm pluteus larval development, postoral arm were further elongated and the postoral and anterolateral arms were supported by well-formed skeletal rods (Figure 2(e)). The digestive tract moved centrally through the larva. The mouth, the clear opening at the base of the shorter (anterirolateral) arms, was followed by a constricted mscular esophagus, which exhibited peristalsis during feeding. Posterodorsal arms, the third pair were first appeared to form this stage (Figure 2(e)). The darkened appearance of the bulbous stomach section is due to the concentration of engulfed algae (Figure 2(e)).

In 8-arm pluteus larva, postetodorsal arms further elongated and the preoral arms, the fourth pairs of arms appeared to form after 16.00 d of fertilization (Table 2; Figure 2(f)). Arched pigmented ciliated bands between the postoral arms began to develop. Immediately above the bands was the mouth cavity enclosed on the lower surface by a small concave edged lobe and on the upper surface by a largest overhanging fold, from which two small preoral arms was protruded. These preoral arms were the fourth pair of larval arms (Figure 2(f)). In this event, characteristic thorns of the skeletal rods were also visible in the postoral arms. 

The precompetent (premature) larval stage began to form 28.00 d after fertilization (Table 2). In this stage, the basal portion of the larva was enlarged and the pigmented arches appeared to form and the pedicellaria was encircled with a ciliated ring (Figure 2(g)). Increased differentiation of adult tissue accounted for the dense appearance of the interior portion of the larva.

The rudiment developed tubefeet and spines, which became active still inside the larval body (Figure 2(h)). No pedicellariae were formed on the surface of the larval body, as commonly observed in competent larvae of regular echinoids. A continued degeneration of larval tissue and arms accompanied by the emergence of the adult spines and tubefeet may be seen slightly below the left corner of the larva. Under the temperature of 24°C, this stage was reached at approximately 35 days (Table 2). 

#### 3.2.1. Metamorphosis

Competent larvae exhibited a typical substrate-test behavior which consisted of swimming near the bottom. In this stage, well-formed spines and extended tubefeet were evident. Larval structures were discarded or absorbed at this point (Figure 2(i)). Metamorphosis occurred when larvae attached firmly to the bottom with the protruding tubefeet and the larval tissues began to regress and accumulate on the aboral surface of the rudiment. During this process, larval spicules became exposed and broke off and the larval tissues accumulated on the aboral surface of the rudiment forming a globoid structure. Metamorphosis took approximately 1 h 30 min from attachment to the complete regression of the larval tissues. 

#### 3.2.2. Juvenile

Metamorphosis was followed by the resorption of larval tissues and the development of complete juvenile structure with adult spines, extended tubefeet and well-developed pedicellaria (Figure 2(i)) and the whole event usually took place within 1 d after settlement (Table 2). Early postlarval juveniles had no skeleton on the aboral surface, except for the remnants of larval rods. The gut was not yet formed and neither mouth nor anus was present. During the resorption of larval tissues, the rudiments of Aristotle's lantern and teeth were visible in the oral region under polarized lights.

## 4. Discussion

Cleavages and development of embryo and larva of *S. sphaeroides* were similar to those reported in other echinoids with planktotrophic larvae [33–38]. The developmental timing of hatching blastulae took longer period (08.45 h at 24°C) than those in *Lytechinus variegatus* (6 h at 23°C) [34] and in *Clypeaster subdepressus* (7 h at 26°C) [38]. Developmental timing of later stages followed the same trends but slightly differed from those of Caribbean species of *L. variegatus* at 23°C [34] and the Pacific species of *Colobocentrotus mertensii* at 27°C [37]. 

Gastrulaion robusts with the correlation between the types of gastrulation and the pattern of migration of red-pigmented cells in *S. sphaeroides*. Red-pigmented cells originate on the vegetal pole and migrate through the ectoderm to the apical plate while the archenteron elongation is continuous. Similar phenomena were observed during the onset of gastrulation in tropical sea urchin *Echinometra mathaei* [39] and the sea biscuit *Clypeaster subdepressus* [38]. Red-pigmented cells can have regulatory role and are known to trigger gastrulation in *E. mathaei* [39]. These cells might participate on the morphological changes occurring during the formation of prism and early axis specification of pluteus larvae [37, 39]. Additionally, the triradiate spicules, the first sign of larval skeleton, were formed during gastrulation in *S. sphaeroides*, which were more or less similar to those observed in other regular echinoids [37, 38].

Competent larvae of *S. sphaeroides* demonstrated substrate-test behavior similar to those documented in other echinoid species [38, 40–43]. Although early postlarval juveniles resemble regular urchins with a spherical body, bilateral symmetry could be identified soon after the resorption of larval tissues and was probably determined during rudiment formation as those observed recently in sea biscuits [38]. In newly metamorphosed juvenile of *S. sphaeroides* (Figure 2(i)), larval arms were completely absorbed together with the skeletons and epidermis. On the contrary, in *Eucidaris thouarsi* [44] and *Paracentrotus lividus* [42], tissue resorption is achieved by the retraction of only epidermis resulting in the naked skeleton. The naked skeletal rods will eventually be broken down. Such type of discrepancy may be related to the species differences [37].

Following the induction of complete metamorphosis, *S. sphaeroides* juveniles had 4 primary spines per interambulacrum (20 totals), similar to those documented in *P. lividus* [42] and *Strongylocentrotus purpuratus* [45]. The irregular echinoid *Echinocardium cordatum* has a greater number of primary spines per interqambulacrum after metamorphosis and also differs from *S. sphaeroides* by the presence of secondary spines and a subanal facsciole and 4 primary spines [43]. Similar to the events in *S. fanciscanus* and *S. purpuratus* [45], *P. lividus* [42], and *E. cordatum* [43], the newly metamorphosed juveniles of *S. sphaeroides* had one tubefoot per ambulacrum. In contrast, *C. subdepressus* uniquely displayed three podia after metamorphosis [38]. Competent larvae of *S. sphaeroides* have pedicellariae during the late larval period and after metamorphosis as those documented in other regular urchins, *P. lividus* [42] and *S. fanciscanus* [45], while pedicellariae of *S. purpuratus* appeared some time after metamorphosis. On the contrary, competent larvae of *E. cordatum* do not exhibit spines or pedicellariae [43], while those of *C. subdepressus* do have spines but devoid of any pedicellariae [38]. 

The young juvenile of *S. sphaeroides* has neither a mouth nor anus and no guts either. Similar event was also observed in other sea urchins [37, 46, 47] and sea biscuits [33, 38]. At this stage, the dorsal half is essentially a rounded lump of larval tissue punctured by the three pedicellaria. The dorsal organs appear to develop out of this tissue. For the first 2 days, the larval tissue can easily be picked off the urchin. The digestive system and probably other internal organs appeared at about 4-5 days after settlement and then the urchin began to feed, as similar to those documented in *Colobocentrotus mertensii* [37], *P. lividus* [42], and *L. pictus* [46]. In contrast, the juveniles of *C. subdepressus* and *C. rosaceus* start feeding 7 and 10 days, respectively, when the Aristotle's lantern and mouth become functional [33, 38]. However, the consequences of these developmental basis in progressing juvenile stages of *S. sphaeroides* deserve further investigations. 

 In conclusion, this study demonstrates the first successful investigation on the embryonic, larval, and postmetamorphic juvenile development of *S*.* sphaeroides* from Peninsular Malaysia. The findings obtained from the designated study would immensely be helpful towards the understanding of ontogeny and life-history strategies, which will eventually assist us in the development of breeding, larval rearing, seed production, and culture techniques of sea urchins for aquaculture industry. 

## Figures and Tables

**Table 1:** Embryonic developmental events of *S. sphaeroides*. Three replicate fertilization experiments were conducted and for each developmental stage, 10 embryos from each replicate were used for the observation and measurement of embryos.

Time after insemination	Developmental stages	Diameter (*μ*m)
00.01 h	Fertilized eggs with the formation of fertilization membrane	134.86 ± 5.35
00.05 h	Fertilized eggs with complete fertilization membrane	134.86 ± 5.35
01.12 h	2-cell stage	154.27 ± 7.17
02.03 h	4-cell stage	157.14 ± 5.84
02.28 h	8-cell stage	159.25 ± 6.29
02.51 h	16-cell stage	161.10 ± 5.80
03.12 h	32-cell stage	163.65 ± 4.78
03.32 h	Multicell (Morulla) stage	164.38 ± 4.48
08.45 h	Hatching Blastula	174.72 ± 4.43

**Table 2:** Larval developmental events of *S. sphaeroides*. Three replicate fertilization experiments were conducted and for each developmental stage, 10 embryos from each replicate were used for the observation and measurement of larvae.

Time after insemination	Developmental stages	Length (*μ*m)
16.15 h	Gastrula	178.71 ± 5.52
22.25 h	Prism	181.56 ± 3.99
34.00 h	2-arm pluteus	233.01 ± 10.51
48.00 h	4-arm pluteus	364.72 ± 6.57
10.00 d	6-arm pluteus	545.62 ± 10.72
16.00 d	8-arm pluteus	716.85 ± 9.99
28.00 d	Precompetent larva with ciliated ring and growing rudiment	929.02 ± 9.62
35.00 d	Competent larva with complete rudiment	740.36 ± 11.51
36.00 d	Juvenile (1 d after metamorphosis)	413.98 ± 6.11
